# Differential expression of microRNAs in the hippocampi of male and female rodents after chronic alcohol administration

**DOI:** 10.1186/s13293-020-00342-3

**Published:** 2020-11-23

**Authors:** Mi Ran Choi, Jasmin Sanghyun Han, Yeung-Bae Jin, Sang-Rae Lee, In Young Choi, Heejin Lee, Hyun Cho, Dai-Jin Kim

**Affiliations:** 1 Department of Psychiatry, Seoul St. Mary’s Hospital, The Catholic University of Korea College of Medicine, 222 Banpo-daero, Seocho-gu, Seoul, 06591 Republic of Korea; 2 National Primate Research Center, Korea Research Institute of Bioscience and Biotechnology, Cheongju, Chungbuk, 28116 Republic of Korea; 3 Department of Medical Informatics, College of Medicine, The Catholic University of Korea, 222 Banpo-daero, Seocho-gu, Seoul, 06591 Republic of Korea; 4 Department of Biomedicine & Health Sciences, College of Medicine, The Catholic University of Korea, 222 Banpo-daero, Seocho-gu, Seoul, 06591 Republic of Korea

**Keywords:** Alcohol, Doublecortin, Hippocampus, MicroRNA, Sex differences

## Abstract

**Background:**

Women are more vulnerable than men to the neurotoxicity and severe brain damage caused by chronic heavy alcohol use. In addition, brain damage due to chronic heavy alcohol use may be associated with sex-dependent epigenetic modifications. This study aimed to identify microRNAs (miRNAs) and their target genes that are differentially expressed in the hippocampi of male and female animal models in response to alcohol.

**Methods:**

After chronic alcohol administration (3~3.5 g/kg/day) in male (control, *n* = 10; alcohol, *n* = 12) or female (control, *n* = 10; alcohol, *n* = 12) Sprague-Dawley rats for 6 weeks, we measured body weights and doublecortin (DCX; a neurogenesis marker) concentrations and analyzed up- or downregulated miRNAs using GeneChip miRNA 4.0 arrays. The differentially expressed miRNAs and their putative target genes were validated by RT-qPCR.

**Results:**

Alcohol attenuated body weight gain only in the male group. On the other hand, alcohol led to increased serum AST in female rats and decreased serum total cholesterol concentrations in male rats. The expression of DCX was significantly reduced in the hippocampi of male alcohol-treated rats. Nine miRNAs were significantly up- or downregulated in male alcohol-treated rats, including upregulation of miR-125a-3p, let-7a-5p, and miR-3541, and downregulation of their target genes (*Prdm5*, *Suv39h1*, *Ptprz1*, *Mapk9*, *Ing4*, *Wt1*, *Nkx3-1*, *Dab2ip*, *Rnf152*, *Ripk1*, *Lin28a*, *Apbb3*, *Nras*, and *Acvr1c*). On the other hand, 7 miRNAs were significantly up- or downregulated in alcohol-treated female rats, including downregulation of miR-881-3p and miR-504 and upregulation of their target genes (*Naa50*, *Clock*, *Cbfb*, *Arih1*, *Ube2g1*, and *Gng7*).

**Conclusions:**

These results suggest that chronic heavy alcohol use produces sex-dependent effects on neurogenesis and miRNA expression in the hippocampus and that sex differences should be considered when developing miRNA biomarkers to diagnose or treat alcoholics.

**Supplementary Information:**

The online version contains supplementary material available at 10.1186/s13293-020-00342-3.

## Background

Recently, the incidence of alcohol use disorders (AUDs) has increased in women as well as men, and AUDs have increased rapidly in young women worldwide, especially in South Korea [1]. This increase is thought to be due to the fact that the perception of women’s traditional roles is gradually changing, and as women participate more broadly in society, their opportunities to drink alcohol have increased [2]. Body fat interferes with dehydrogenation of alcohol, and the body fat percentage is greater in women than in men; women also have a relatively lower percentage of water in their bodies than men [3].Due to these characteristics, alcohol metabolism in women is less efficient than in men, suggesting that women are more vulnerable to alcohol-induced liver damage than men. In addition, women are more vulnerable to neurotoxicity, volumetric brain atrophy, and cognitive dysfunction induced by alcohol than men [1, 4–6].

In animal models of short-term binge alcohol exposure in both sexes, the numbers of neurons in the hippocampi were reduced only in female rats, showing that binge alcohol drinking produces greater negative effects in the hippocampi of females [7]. Previous studies have indicated that women’s AUDs have become increasingly important; however, to date, treatment of AUDs in most countries including South Korea and the USA has mainly focused on men, and laboratory studies on the negative effects of alcohol have typically used only male animals.

The hippocampus is located in the limbic system, which controls memory and emotions. It is composed of the dentate gyrus (DG) and the cornu ammonis (CA), the latter of which comprises 4 regions (CA1, CA2, CA3, and CA4) [8]. The hippocampus is involved in cognitive functions and is vulnerable to toxic reagents such as alcohol at all life stages, including during fetal development. Many studies involving animal models or human exposures have reported that alcohol causes neurotoxicity and impairs neurogenesis and neural plasticity in the hippocampus, and consequently produces cognitive dysfunction [9–13]. Doublecortin (DCX), a brain-specific microtubule-associated protein, is known as a neurogenesis marker and plays an important role during migration of immature neurons and neurite development in the hippocampus [14, 15]. Some studies have demonstrated that following binge alcohol exposure for 4 days, DCX expression was reduced in the DG of the hippocampus of rats, similar to reported post-mortem findings in the hippocampal DG of alcohol abusers, suggesting that excessive alcohol use inhibits neurogenesis in the hippocampus regardless after both short-term high-dose exposure or long-term drinking [16–18]. However, studies addressing how chronic exposure to the same amount of alcohol affects neurogenesis in the hippocampus differently in males and females are currently lacking.

MicroRNAs (miRNAs), which are small noncoding RNA molecules of 20–25 nucleotides, are expressed in various tissues including the hippocampus. miRNAs regulate various physiological and pathological processes, such as cell proliferation, immunity, and tumorigenesis, by cleavage, translational repression, and deadenylation of specific mRNAs [19, 20]. Recently, it has been reported that changes in miRNAs and their target genes induced by alcohol exposure may affect brain function [21–23]. A previous study identified alterations of miRNA expression and their associated gene networks in the amygdala (AMY), nucleus accumbens (NAc), and prefrontal cortex (PFC) of the mouse brain after chronic alcohol exposure, suggesting correlations between alterations of miRNA and changes in brain function induced by alcohol [24]. AUDs or frequent heavy drinking of alcohol induced alterations in miRNAs, such as miR-9 and miR-124, and their target genes in the striatum, resulting in changes of sensitivity to alcohol [25, 26]. In addition, a previous study reported that chronic intermittent alcohol exposure increased miR-206 expression and decreased that of its target gene BDNF in the hippocampi of male mice [27]. However, to date, studies indicating that alcohol affects the expression of miRNAs and their target genes in the brain have been limited to the male sex.

In consideration of these findings, we hypothesized that alcohol may produce sex-dependent effects on neurogenesis and miRNA expression. Sex-dependent effects on neurogenesis and epigenetic regulation caused by alcohol suggest that sex differences should be considered when developing biomarkers for alcoholism or alcohol-related cognitive impairments. In this study, we evaluated changes in body weight, memory performance, DCX, and biochemical parameters in male and female rats. In addition, we performed miRNA expression profiling and investigated the expression of miRNA target genes in the hippocampus of male and female rats and identified significant changes in the expression of 9 miRNAs, including miR-125a-3p, in male rats, and 7 miRNAs, including miR-881-3p in female rats.

## Methods

### Animals

Forty-four 6-week-old male and female Sprague-Dawley (SD) rats were purchased from Central Lab Animal Inc. (Seoul, Korea) and underwent a 1-week period of adjustment to the experimental environment. The rats were weighed and randomly divided into two groups per sex as follows: male control (MC, *n* = 10) and alcohol-treated group (MA, *n* = 12) groups; female control (FC, *n* = 10) and alcohol-treated (FA, *n* = 12) groups. The rats were housed on a 12-h:12-h light:dark cycle at constant room temperature (22 ± 1 °C) throughout the experiments.

### Alcohol exposure

All experimental groups were group housed with ad libitum access to food and water during the experiments. The 7-week-old control and alcohol-treated groups were orally administered 0.9% saline or 3–3.5 g/kg ethanol through gavage, respectively, once daily at 10:00 AM, as described previously [28]. The body weights of rats were measured once a week, and the total amount of ethanol was increased in proportion to the increase in body weight in order to maintain a constant dose on a mg/kg basis. After two female alcohol-treated rats died in the first week of daily administration of 3.5 g/kg ethanol, the ethanol doses were reduced to 3 g/kg from week 2 onward. When dead female rats were autopsied, the lungs were bloodshot, the gastric walls were thinned, and the gastric mucosa was inflamed with ulcers. The livers were dark red and enlarged, showing damage due to alcohol. Ethanol administration was continued for 6 weeks. The numbers of animals that survived throughout the experiment were as follows: MC, *n* = 10; MA, *n* = 12; FC, *n* = 10; FA, *n* = 7.

### Radial arm maze test

At the 6th week of alcohol administration, the radial arm maze test was performed to evaluate learning and memory performance. All experiments were carried out in dimly light room with visual cues that were fixed on the walls of the room during the experiment forming spatial references for the animal during the learning and memory phases. The male and female rats were deprived of food for 10 h prior to the radial arm maze test to induce hunger; after which, they were moved to the radial arm maze and were trained to find food pellets located at the end of the arm. First, we placed them in the center of the radial arm maze, allowed them to adapt for 60 s, and then opened the entrance into each arm at the same time. The experiment was concluded when the animal visited one arm 16 times within 15 min or at 15 min after the experimental onset, regardless of number of visits. These training procedures were repeatedly performed over 3 days. Whenever a rat entered a new arm among the 8 total, it was able to obtain a food pellet; but if the rat entered the same arm repeatedly, no food pellet was available. On the 4th day, to measure working memory error of rats due to chronic alcohol exposure, we recorded the number of times that the rat entered arm over a 15-min period and recorded an error when the rat entered the same arm more than two times. To evaluate working memory error of rats due to chronic alcohol exposure, we calculated the average re-entry number of 7 rats in each group.

### Serum biochemistry

After the male and female rats were administered alcohol or vehicle for 6 weeks, they were anesthetized with isoflurane (1–2% with 0.8 L/min O_2_ administered with a facemask), and 3–5 mL of blood was drawn directly from the heart. Blood samples were centrifuged at 800*g* for 20 min at 22 °C, and the upper phase was transferred into a fresh tube. The serum biochemical parameters were measured by SCL Healthcare (Yongin, Korea).

### Immunohistochemistry

Hippocampal tissues dissected from the brains of the MC, MA, FC, and FA groups were fixed with 4% formaldehyde for 24 h, washed with distilled water and dehydrated gradually with a series of 70–100% ethanol washes. The tissues were immersed in xylene, embedded in paraffin, and sliced into 3-μm sections. The sections were transferred onto slides, and the slides were deparaffinized with xylene. The slides were hydrated through graded alcohol washes into water. For antigen retrieval, the slides were heated at 95 °C for 20 min in Dako^TM^ Target Retrieval Solution, pH 6.0 (Dakocytomation, Carpinteria, CA, USA). After cooling for 20 min, the slides were quenched with 3% H_2_O_2_ for 5 min. The slides were then incubated with rabbit anti-DCX antibody (1:3000) (ab18723, Abcam, Cambridge, MA, USA) for 2 h at room temperature. Endogenous peroxidase was blocked using DAKO REAL peroxidase blocking solution (Dakocytomation, Carpinteria, CA, USA) for 10 min. Antibody was detected using DAKO EnVision+ for rabbit antibody (K4003, DAKO, Glostrup, Denmark) for 1 h, and signal was detected with a Dako REAL^TM^ DAB+ Chromogen detection system (Dakocytomation, Carpinteria, CA, USA) according to manufacturer’s instructions. The slides were counterstained with hematoxylin.

Slides were scanned using a Panoramic MIDI scanner (3DHISTECH Ltd, Budapest, Hungary). Two to three samples within each group and two slides per sample were scanned for density measurements. Digital image analyses of each slide were performed at × 200 magnification with the Panoramic Viewer and HistoQuant software (3DHISTECH Ltd, Budapest, Hungary). The DCX expression intensity in the DG area of the hippocampus region was then measured as the H-score using the HistoQuant tool in the Panoramic Viewer (3DHISTECH Ltd., Budapest, Hungary) with the positive pixel-counting algorithm, which scores the stains as negative, weak-positive, medium, and strong [29, 30]. This algorithm established the H-score of each case based on percentage and intensity of staining. The H-score considers the intensity of staining and the percentage of positive cells in the formula: H-score = 1 × (% light staining) + 2 × (% moderate staining) + 3 × (% strong staining). H-score values of samples in each group were calculated, analyzed statistically, and presented as a graph.

### Microarray analyses

Total RNA was extracted from tissues using Trizol™ reagent (Thermo Fisher Scientific, IN, USA). The extracted RNA was labeled using the FlashTag^TM^ biotin HSR RNA labeling kit (Affymetrix Inc., Santa Clara, CA, USA) according to the manufacturer’s instructions. Briefly, 800 ng of RNA was poly-A tailed, and a proprietary biotin-labeled dendrimer molecule was joined to the 3’end using DNA ligase. The biotin-labeled samples were hybridized to GeneChip miRNA 4.0 microarrays (Affymetrix Inc., Santa Clara, CA, USA). A hybridization mixture with controls, including oligo B2, 20X hybridization controls (bioB, bioC, bioD, and cre), 27.5% formamide, DMSO, 2X hybridization buffer, and water, was added to all the samples. The hybridization mixture was injected into GeneChip miRNA 4.0 arrays (Affymetrix, Santa Clara, California, USA), and the arrays were placed in the Affymetrix GeneChip Hybridization Oven 640 at 48 °C and 60 rpm for 16 h overnight. Stain cocktails (stain cocktail 1 and stain cocktail 2) were added to amplify signal intensities. Arrays were stained and washed in the Affymetrix GeneChip Fluidics Station 450. All arrays were scanned with the Affymetrix GeneChip Scanner 3000 and analyzed using the Transcriptome Analysis Console^TM^ software. The raw data images produced from the scanner were processed into CEL files, which contained measured intensities for each probe on the array. The Signal Space Transformation-Robust Multiarray Average algorithm was applied to background-adjust, normalize, and log-transform the signal intensity values. The signal intensity ratio of the alcohol-treated group (*n* = 4) compared to the control group (*n* = 4) for each sex was converted into a log value and then converted into a fold change, and the expression change value was calculated. The signal intensity ratio of alcohol-treated group compared to the control group in each sex was converted into a log value, and the expression levels were obtained by converting the log ratio into a fold change. The expression of each miRNA in the alcohol-treated groups relative to the control group in each sex was validated by applying the unpaired *t* test (*p* < 0.05). miRNAs for which there was a greater than 1.5-fold difference between the control and alcohol-treated groups were selected for further analyses.

### Identification of miRNA target genes and gene annotation enrichment

Target genes of significantly up- or downregulated miRNAs in the alcohol-treated groups in each sex were identified using TargetScan 7.2 and miRDB resources. Genes identified in both TargetScan and miRDB were selected, and their functional annotation was performed using the DAVID 6.8 tool (https://david.ncifcrf.gov). Statistically over-represented gene ontology (GO) categories at *p* < 0.05 were considered significant. GO categories were classified into three subcategories (biological process (BP), cellular component (CP), and molecular function (MF)) and the KEGG pathway. Of the GO categories, several genes belonging to the BP subcategory and KEGG pathway were selected for further analyses.

### Quantitative reverse transcription PCR

To validate the miRNAs differentially expressed in the microarrays and the expression levels of some target genes, we performed quantitative reverse transcription PCR (RT-qPCR). For miRNAs, total RNA was extracted from the hippocampi of MC (*n* = 4), MA (*n* = 4), FC (*n* = 4), and FA (*n* = 4) groups using TRIzol™ Reagent and PureLink™ miRNA isolation kits (Thermo Fisher Scientific, San Jose, CA) and reverse transcribed to cDNA using TaqMan^TM^ MicroRNA reverse transcription kits according to the manufacturer’s instructions. TaqMan MicroRNA assays (Thermo Fisher Scientific, San Jose, CA) were used to perform qPCR for miRNAs (rno-miR-125a-3p, rno-let-7a-5p, rno-miR-3541, rno-miR-881-3p, and rno-miR-504) and U6 snRNA (internal control) according to the manufacturer’s instructions. The qPCR experiments were performed as three independent replicates to guarantee reliable results for all samples. Differential expression of miRNAs in each sample was normalized to U6 snRNA expression. The magnitude of the changes in the expression of differentially expressed miRNAs was calculated using the 2^−ΔΔCT^ method. For target genes, total RNA was extracted from the hippocampi of the MC (*n* = 4), MA (*n* = 4), FC (*n* = 4), and FA (*n* = 4) groups using TRIzol™ Reagent (Thermo Fisher Scientific, San Jose, CA) and reverse transcribed to cDNA using the Superscript™ RT III system (Thermo Fisher Scientific, San Jose, CA). Details of the methods for qPCR have been described previously [31]. The qPCR experiments were performed as three independent replicates to guarantee reliable results for all samples in each group (MC, MA, FC, and FA groups). Differential expression of genes in each sample was normalized to GAPDH expression. The differences in relative gene expression between the MC and MA groups and FC and FM groups were calculated using the 2^−ΔΔCT^ method. The primers used for the amplification of candidate genes are presented in Table 1.

**Table 1:** Primers used in RT-qPCR

Gene	Forward (5′-3′)	Reverse (5′-3′)	Amplicon size (bp)
*Gapdh*	TTACCAGGGCTGCCTTCTCTT	TTGAACTTGCCGTGGGTAGAGT	118
*Prdm5*	TGGGACCCTGAGAGTTCACATC	TCCGTCATTTTTACTGAATCCTTTT	91
*Suv39h1*	GCGACTCTAGGTTGCAGTGTGT	AAGGGCAGGACAAGAAAGCTT	90
*Ptprz1*	GGGTTAAGCTCACACAACTTGCT	TGTAAGCCTTTGGCCTGTTGTA	96
*Mapk9*	CACGGACAGCCTGTACCAACT	TGTAGCCCATGCCCAGGAT	90
*Ing4*	TTGGCTGTGACAACCCAGACT	GCGTGGGCAAAACCATTTC	95
*Wt1*	ACGGCACAGGGTACGAGAGT	CCTGAATGCCTCGGAAGACA	96
*Nkx3-1*	GGCTCACCTGGCCAAGAAC	CTGACAGCTGCCTTCGCTTA	95
*Dab2ip*	ACCCAGTGGTGACACCTAATCC	GCAAGATGGTGATGGTCTGGTA	90
*Rnf152*	CACACCCCAGTCTTCATCAAAC	GTCTCCCGGCAGGAGTGTAC	90
*Ripk1*	TCAAGCCGTCTTCGCTAACA	TTCGGGCACAGTTTTTCCA	95
*Lin28a*	CCTGGTGGCGTGTTCTGTATT	GTTGTAGCACCTGTCTCCTTTGG	90
*Apbb3*	CTCATCGCTGATCTTGGTTGTC	CAGGCAGCTTGCACAGCTT	95
*Nras*	CGAAGGGTTCCTCTGTGTGTTT	TCTTTCACGCGCTTAATTTGC	90
*Acvr1c*	GGTATATGGCTCCCGAAATGC	GTAAACCAGCCCCACCGAAT	95
*Naa50*	GTCAGCGATTGACTTCTACAGGAA	TGAGGCTTTTCTGAAGCACATG	110
*Clock*	AGCTGCTGACAAAAGCCAAGA	AGGAGTTGGGCTGTGATCGA	97
*Cbfb*	TTGAAAGCCCCCATGATTCT	CTCCAGGCAACCCATACCAT	90
*Arih1*	TCGGGCTACCTTGAACGAGAT	AAACCCTTCGTCGACTCTCACA	100
*Ube2g1*	ACGCTGGCTGCCTATCCATA	TCCATTCTTTCGCAGCATCTAC	110
*Gng7*	GGAAGCTGGTGGAGCAGTTG	CGGGCATGTTGCTCACAGTA	100

### Statistical analyses

Statistical analyses were conducted with the SPSS® 18.0 software (SPSS Inc., Chicago, IL, USA) and GraphPad Prism 8 software (San Diego, CA, USA). Comparisons of biochemical factors, DCX intensity, and expression of miRNAs and their target genes between control and alcohol-treated groups in each sex were performed using two-sample *t* tests and Mann-Whitney *U* tests for normally distributed variables and non-parametric variables, respectively. All data were expressed as the means ± standard error of the mean (SEM). *P* < 0.05 was considered statistically significant. The repeated measured body weights were compared among sexes and alcohol-treated groups using two-way repeated measures ANOVA.

## Results

### Alteration of body weights and working memory due to chronic alcohol administration

**Fig. 1:**
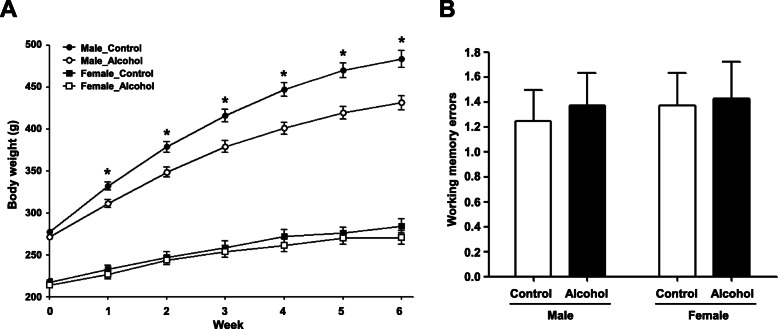
Body weights and behavioral action test. **a** Body weights of male (control, *n* = 10; alcohol, *n* = 12) and female (control, *n* = 10; alcohol, *n* = 7) rats during alcohol administration. **b** The effect of alcohol on working memory. Re-entry number is the number of times the rat entered the same arm among the eight arms (7 rats were tested in each group). *Significantly different from male control group (*p < 0.05)

When we measured body weights of the control (MC and FC) and alcohol-treated (MA and FA) groups, male rats had greater body weight gains than female rats during alcohol administration period (Fig. 1a). When comparing changes in body weight between the control and alcohol-treated groups of each sex, the body weight gain in the MC group was significantly greater than that in the MA group from week 1 to week 6, while there were no differences in body weight gain between the FC and FA groups throughout the period of alcohol administration. A two-way repeated measures ANOVA (treatment × sex × weight, with repeated measures on weight) revealed a significant treatment × sex × weight interaction [*F*(*df* = 1.976) = 7.026, *p* = 0.002]. These results suggest that chronic alcohol exposure affected body weight gain differently in males and females. On the other hand, when comparing the re-entry error rate between control and alcohol-treated groups in each sex, there were no significant differences between control and alcohol-treated groups (Fig. 1b).

### Alterations in biochemical parameters and lipid expression after chronic alcohol administration

To identify the effects of alcohol in the MC, MA, FC, and FA groups, we measured the serum concentrations of factors related to toxicity and lipid metabolism as described previously [29, 32]. Elevated serum amylase concentrations are associated with pancreatitis; there were no significant differences between serum amylase concentrations in the control and alcohol-treated groups of either sex (Table 2), although the FA group had greater amylase concentrations than the FC group (*p* = 0.095). Blood urea nitrogen (BUN) and creatinine concentrations, which are markers of kidney toxicity, did not differ between the control and alcohol-treated groups of either sex. When measuring serum aspartate aminotransferase (AST) and alanine aminotransferase (ALT) concentrations, markers of liver fat accumulation, secretion level of AST did not differ between the MC and MA groups; however, that in the FC group was significantly higher than that in the FA group (Table 2). In contrast, serum ALT concentrations did not differ between the control and alcohol-treated groups of either sex. When assessing effects on lipid metabolism in the alcohol-exposed rats using serum high-density lipoprotein cholesterol (HDLC), total cholesterol (TC), and triglyceride (TG), HDLC was reduced in the MA group, but this change was not statistically significant (*p* = 0.087). In particular, TC in the MA group was significantly lower than in the MC group (Table 2). In contrast, serum HDLC and TC did not differ in the FC and FA groups. Serum TG concentrations did not differ in the control and alcohol-treated groups of either sex, while regardless of alcohol administration, TG concentrations in male groups were greater than in the female groups. When the serum concentrations of gamma-glutamyl transpeptidase (GGT) and total bilirubin were measured, their concentrations were not detected or were detected below normal levels (data not shown).

**Table 2:** Secretion of biochemical parameters and lipids in blood after chronic alcohol administration

Biological factor	Male			Female		
	Control (*n* = 5)	Alcohol (*n* = 5)	*P* value	Control (*n* = 5)	Alcohol (*n* = 5)	*P* value
Amylase (U/I)	2407 ± 191.65	2587 ± 181.14	0.691	1939 ± 121.50	2404 ± 229.22	0.095
BUN (mg/dl)	15.00 ± 0.70	14.80 ± 0.86	0.968	17.00 ± 0.83	16.60 ± 0.74	0.738
Creatinine (mg/dl)	0.27 ± 0.01	0.30 ± 0.01	0.333	0.38 ± 0.01	0.37 ± 0.02	0.651
AST (U/l)	95.80 ± 3.20	103.40 ± 14.94	0.706	121.60 ± 7.88	161.40 ± 11.58	0.047^*^
ALT (U/l)	42.60 ± 3.31	46.60 ± 5.75	0.706	42.20 ± 3.07	40.00 ± 2.94	0.794
HDLC (mg/dl)	69.40 ± 5.67	55.40 ± 6.12	0.087	74.00 ± 6.54	84.20 ± 5.18	0.571
TC (mg/dl)	84.80 ± 4.96	68.80 ± 5.20	0.040^*^	80.20 ± 7.95	89.00 ± 5.47	0.841
TG (mg/dl)	178.80 ± 21.58	172.00 ± 39.99	0.691	100.20 ± 18.89	87.40 ± 13.34	0.421

*BUN* blood urea nitrogen, *AST* aspartate aminotransferase, *ALT* alanine transaminase, *HDLC* high-density lipoprotein cholesterol, *TC* total cholesterol, *TG* triglyceride

^*^Significantly different between control and alcohol-treated groups in each sex (*p* < 0.05)

### Altered expression of DCX in the hippocampi of rats after chronic alcohol administration

**Fig. 2:**
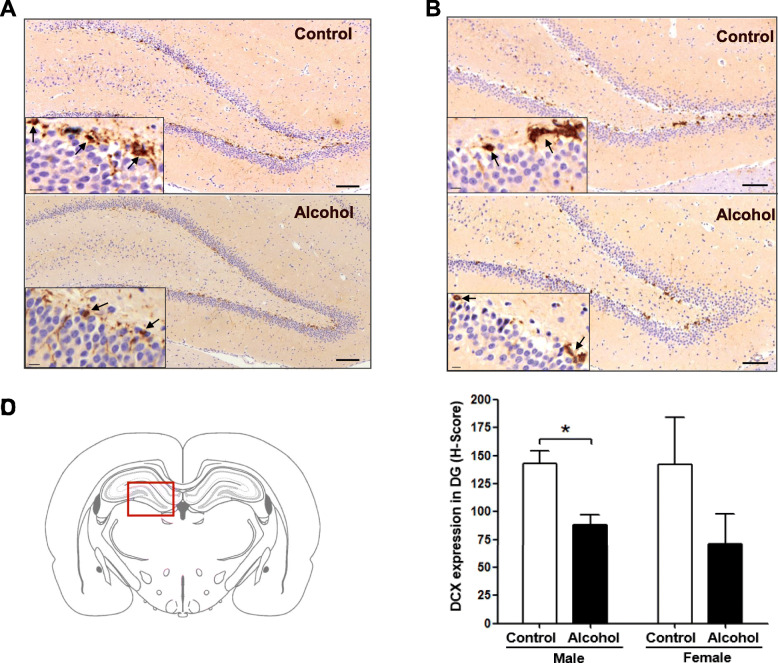
Expression of DCX in the hippocampi of male and female rats. **a** DCX expression in the hippocampi of male rats (*n* = 3). **b** DCX expression in the hippocampi of female rats (*n* = 3). Scale bar = 100 μm (insert: 10 μm). Black arrows indicate high DCX-expressing cells. **c** Rat brain atlas. The red square is the hippocampal dentate gyrus (DG) region where neurogenesis occurs and where DCX expression was measured. **d** The percentage of cells that are positive for DCX is expressed as H-score. *Significantly different from male control group (*p < 0.05)

To investigate the effects of alcohol on neurogenesis, we measured DCX protein expression in the hippocampal DG region where immature neurons express DCX (Fig. 2c). In the MA group, DCX expression was significantly (*p* = 0.031) reduced compared to the MC group (Fig. 2a and d). In addition, DCX expression was reduced in some animals in the FA group, but the differences in DCX expression in the FC and FA groups were not significant *(p* = 0.29) because of individual variations (Fig. 2b and d). Based on these results, DCX expression in male rats appeared to be a more sensitive to alcohol than in female rats.

### Differential expression of miRNAs in male and female rats after chronic exposure to alcohol

**Fig. 3:**
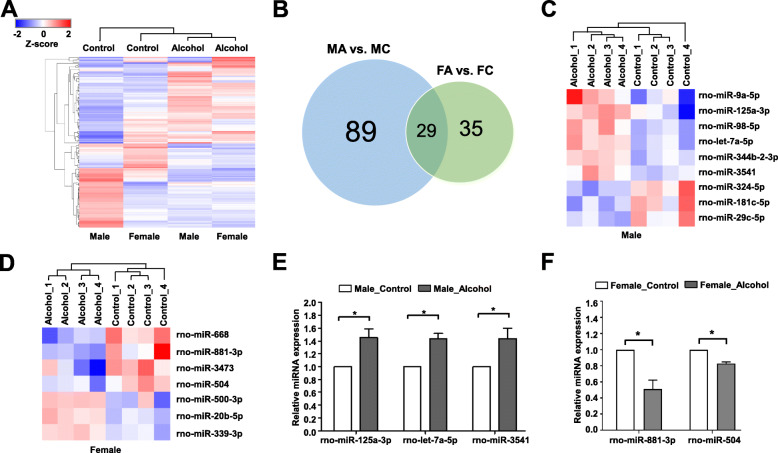
miRNAs expressed differentially between control and alcohol-treated groups. **a** Heatmap described from the two-way hierarchical clustering analyses of differentially expressed (more than 1.5 fold) miRNAs among groups. The hippocampi of four rats in each group were used for the miRNA array. **b** Venn diagram showing the number of differentially expressed miRNAs between two groups. MA, male alcohol-treated group; MC, male control group; FA, female alcohol-treated group; FC, female control group. **c** Comparison of miRNA expression in the male control and alcohol-treated rats. **d** Comparison of miRNA expression in the female control and alcohol-treated rats. **e** Validation of 3 miRNAs upregulated in alcohol-treated male rats (*n* = 4) compared to the control male rats (*n* = 4) using RT-qPCR. **f** Validation of 2 miRNAs downregulated in alcohol-treated female rats (*n* = 4) compared to the control female rats (*n* = 4) using RT-qPCR. *Significantly different from control group in each sex (*p < 0.05)

To investigate sex-dependent expression of miRNAs in the hippocampi of rats in response to alcohol, we performed miRNA expression profiling using microarrays. After log2 transformation of the magnitude of miRNA up- or downregulation (fold change cutoff of 1.5 as compared to the control groups), a hierarchical heatmap of 153 miRNAs was constructed (Fig. 3a). Of 153 differentially expressed miRNAs, 89 were differentially expressed in male rats only, while 35 were differentially expressed in female rats only (Fig. 3b and Supplementary Table 1). In addition, 29 miRNAs were upregulated or downregulated by alcohol in both sexes. Of 118 miRNAs differentially expressed in male rats, 6 miRNAs including miR-125a-3p were significantly upregulated in the MA group, while 3 miRNAs including miR-324-5p were significantly downregulated in the MA group (Fig. 3c). In female rats, of 64 differentially expressed miRNAs, 4 miRNAs including miR-881-3p were significantly downregulated in the FA group, while 3 miRNAs including miR-500-3p were significantly upregulated in the FA group (Fig. 3d).

The up- or downregulation of several miRNAs in response to alcohol exposure was validated using RT-qPCR. Of these, miR-125a-3p, let-7a-5p, and miR-3541 expression in male rats was significantly increased by alcohol exposure (Fig. 3e). In contrast, miR-881-3p and miR-504 expression in female rats was significantly reduced by alcohol exposure (Fig. 3f). Therefore, all the validated miRNAs exhibited the same expression patterns in both microarray and RT-qPCR.

### Expression of miRNA target genes after chronic alcohol administration

**Fig. 4:**
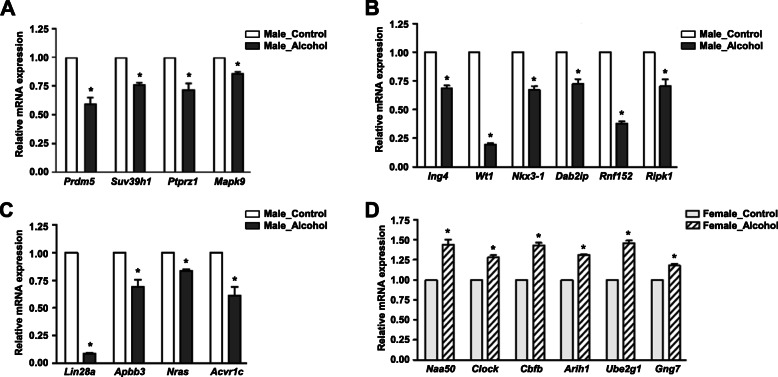
Expression patterns of predicted target genes of miRNAs in male and female rats after chronic alcohol administration. **a** Genes regulated by miR-125a-3p. **b** Genes regulated by miR-3541. **c** Genes regulated by let-7a-5p. **d** Genes regulated by miR-881-3p (*Naa50*, *Clock*, and *Cbfb*) and miR-504 (*Arih1*, *Ube2g1*, and *Gng7*). *Significantly different from control group in each sex (*p < 0.05)

We identified the target genes of differentially expressed miRNAs in male and female rats after alcohol administration and performed GO-based functional annotation of the genes. Of 3 GO terms and KEGG pathway markers classified by functional annotation, genes included in the BP and KEGG pathway categories were selected (Supplementary Table 2 and 3). We performed RT-qPCR to investigate changes in the expression of the target genes of differentially expressed miRNAs. The expression of the target genes of miR-125a-3p (*Pdrm5*, *Suv39h1*, *Ptprz1*, and *Mapk9*) was significantly reduced in the MA group compared to the MC group (Fig. 4a). The expression of the target genes of miR-3541 (*Ing4*, *Wt1*, *Nkx3-1*, *Dab2ip*, *Rnf152*, and *Ripk1*) and let-7a-5p (*Lin28a*, *Apbb3*, *Nras*, and *Acvr1c*) was also significantly reduced (Fig. 4b and c). On the other hand, the expression of the target genes of miR-881-3p (*Naa50*, *Clock*, and *Cbfb*) and miR-504 (*Arih1*, *Ube2g1*, and *Gng7*) increased significantly in the FA group (Fig. 4d). Taken together, the expression of the validated genes was opposite to the expression of the miRNAs that regulate their expression.

## Discussion

As women have increased their participation in all aspects of Korean society, their opportunities to drink alcohol have increased, along with the incidence of AUDs. Women are more vulnerable than men to the neurotoxicity and cognitive dysfunction induced by alcohol because they have less alcohol dehydrogenase enzyme than men [4, 5]. In addition, binge alcohol drinking induces more severe impairment in the hippocampi of women [7]. In view of the fact that alcohol affects the brain, including the hippocampus, differently in males and females, we designed a chronic model of heavy drinking using male and female rats, analyzed the resulting physiological and biological changes in the alcohol-exposed rats, and investigated global miRNA alterations and their target genes in the hippocampus.

In the present study, alcohol did not affect body weight gain in female rats, while alcohol attenuated body weight gain in the male group. In a previous animal study, a single 3 g/kg ethanol injection after injection of 1 g/kg ethanol for 2 weeks reduced appetite and weight gain in male Long Evans rats [33], similar to the findings in male rats in our study. However, another study using voluntary ethanol drinking exposure found no effects of ethanol on weight gain in either sex [33]. In a study by Kolota et al. [34], body weight gain in adolescent (3-week-old) male rats that received solutions of alcoholic beverages (ethanol, red wine, and beer) for 6 weeks was attenuated in all experimental groups, particularly the beer-drinking group, compared to the control group. Our results and those of Nelson et al. [33] suggest that the duration of alcohol administration and weight gain in male rats are inversely proportional; however, further research is needed to address the inconsistency with regard to changes in body weight gain in male rats during voluntary drinking of alcohol. On the other hand, body weight gain in female rats was not affected by alcohol regardless of the method of alcohol exposure. This suggests that alcohol does not disrupt the normal anorectic effect in female rats.

Chronic alcohol abuse damages various tissues including the liver, in particular, as alcohol is primarily metabolized in the liver: the liver may be damaged by reactive oxygen species, cytokines, and chemokines produced during metabolic processes [35]. Liver damage induces AST release into the blood from hepatocytes [36]. In this study, chronic alcohol exposure in female rats induced a significant increase in serum AST concentrations, while AST concentrations were not altered in male rats. Therefore, our results imply that female rats are more vulnerable to liver impairment than male rats when exposed to the same dosage of alcohol for long periods. On the other hand, serum TC in male rats chronically exposed to alcohol was significantly reduced. This finding is consistent with the significant lower body weight gain in male rats chronically exposed to alcohol compared to male control rats. Taken together, the findings of the present study suggest that chronic exposure to about 3 g/kg alcohol leads to more severe liver damage in female rats and lower TC and body weight gain in male rats.

In this study, DCX expression in the hippocampi of male and female rats was reduced by chronic high-dose alcohol administration, with a significant reduction in male rats. DCX, which is highly expressed in immature neurons and is a neurogenesis marker, binds to the microtubule cytoskeleton and stabilizes microtubules [14, 37]. A previous study reported that chronic prenatal alcohol exposure induced significant reduction of DCX expression in the hippocampi of male mouse pups, but not in the hippocampi of female pups [38]. This study suggested that prenatal exposure to alcohol negatively affects neurogenesis in males even after they are born. Postmortem examination of hippocampi from men and women who abused alcohol also found reduced DCX expression compared to the controls, in agreement with our results [16]. Similarly, acute high-hose exposure of male rats to alcohol reduced DCX-positive cells in the hippocampi [17, 39]. Taken together, these findings indicate that alcohol exposure leads to reduced DCX expression in the male hippocampus regardless of the duration of alcohol exposure and drinking age. On the other hand, as there was no change in DCX expression in female pups following prenatal alcohol exposure or few studies that observed DCX expression after exposure to alcohol in females, further studies are needed to determine whether chronic alcohol exposure reduces DCX expression in the female hippocampus.

In the present study, alcohol significantly induced miR-125a-3p and let-7a-5p expression but downregulated their target genes in the hippocampi of male rats. Recently, some papers have reported that miR-125a-3p not only negatively regulates hepatocyte viability and osteoblastic differentiation and proliferation, but also inhibits proliferation and migration of vascular smooth muscle cells [40–42]. Based on these studies, miR-125a-3p is believed to be a negative regulator of proliferation and viability of most cells. Other studies have demonstrated that miR-125a-3p inhibited maturation of oligodendroglial precursor cells derived from rat cerebral cortices [43, 44]. We therefore postulated that chronic alcohol exposure induces upregulation of miR-125a-3p in the male hippocampus, resulting in dysfunction of immature cells. Among many potential targets of miR-125a-3p, we found that *Prdm5*, *Suv39h1*, *Ptprz1*, and *Mapk9* were downregulated in male rats chronically exposed to alcohol. In GO enrichment, these genes are associated with histone H3-K9 methylation, neuron development, oligodendrocyte differentiation, and the foxO signaling pathway. Given methylation of histone lysine residues by methyltransferases (Kmt) regulates nucleosome architecture, the cell cycle, and transcription [45], it has been postulated that downregulation of the Kmt orthologues *Prdm5* and *Suv39h1* by miR-125a-3p after alcohol exposure may also affect the expression of genes associated with the cell cycle and transcription in the hippocampus. In addition, chronic alcohol exposure is known to downregulate the *Ptprz1* and *Mapk9* genes associated with neuron development, oligodendrocyte differentiation, and the foxO signaling pathway, and increased miR-125a-3p expression in the hippocampus by alcohol is thought to regulate these pathways by inhibiting the expression of *Ptprz1* and *Mapk9*.

The expression of let-7a-5p has been reported to be altered in various cancers, including lung [46], colon [47], head [48], breast [49], and pancreatic cancer [50]. In particular, let-7a-5p is known to participate in oncogene expression or carcinogenesis in these cancers. Apart from cancer-related research, increased let-7a-5p was recently found to inhibit osteogenesis of bone marrow-derived mesenchymal stem cells in osteoporotic female mice [51]. Another study reported that let-7a-5p was upregulated in brain microvascular pericytes of spontaneously hypertensive rats, suggesting the possibility of correlations between let-7a-3p and the pathogenesis of hypertension [52]. In the present study, alcohol exposure induced increase of let-7a-5p and decrease of its target genes (*Lin28a*, *Apbb3*, *Nras*, and *Acvr1c*) in the hippocampi of male rats. Considering that among the validated target genes of let-7a-5p, *Nras* and *Acvr1c* are associated with signaling pathways regulating the pluripotency of stem cells, our findings suggest that alcohol may negatively affect differentiation of neural stem cells by inhibiting the expression of its target genes through let-7a-5p.

In this study, chronic alcohol administration reduced miR-881-3p and miR-504 expression, but upregulated their target genes in the hippocampi of female rats. To date, only a few studies have investigated miR-881-3p. A previous study identified upregulation of miR-881-3p in liver tissues of aged rats with non-alcoholic fatty liver diseases [53]; these findings are inconsistent with the downregulation of miR-881-3p in the hippocampi of female rats in our study, although the tissues examined were different and not exposed to alcohol. In another study, male rats chronically exposed to methamphetamine (METH) had lower expression of miR-881-3p in the NAc than those acutely exposed to METH [54]. Taken together, the patterns of miR-881-3p expression arising from exposure to toxic reagents and the occurrence of disease due to aging appear to be opposite, but further investigations are necessary to confirm these findings. However, we identified increased expression of target genes related to histone acetylation (*Naa50* and *Clock*) and protein polyubiquitination (*Cbfb*) together with decreased expression of miR-881-3p. Based on our observations that epigenetic modification-related genes increased after prolonged alcohol exposure, chronic heavy alcohol use may affect epigenetic modification by inhibiting miR-881-3p in the hippocampi of females.

Recently, miR-504 was reported to function as a tumor suppressor by inhibiting cancer cell proliferation and invasion and is downregulated in several cancer types, including hepatocellular carcinoma [55], glioma [56], glioblastoma [57], and hypopharyngeal squamous carcinoma [58]. In the present study, chronic high-dose alcohol exposure downregulated miR-504 expression in female rats with no change in expression in male rats. Therefore, considering that glioma development causes impairment of neuronal cells in the brain, reduction of miR-504 in the hippocampi of female rats chronically administered alcohol suggests that the same dose of alcohol has greater adverse effects on brain cancer-related miRNA expression in female rats than in male rats. In addition, as a result of analyzing changes in the expression of miR-504 target genes, we identified upregulation of *Ar1h1* and *Ube2g1* genes related to protein polyubiquitination, and *Gng7* associated with glutamatergic synapses in the hippocampi of female rats exposed to alcohol. However, as there have been no studies addressing miR-504 and its target-gene-related functions in normal cells, further studies addressing how miR-504 and its target genes in normal cells are influenced by toxic reagents including alcohol are needed.

In conclusion, we found that chronic high-dose alcohol administration reduced body weight gains in male rats but not in female rats. In addition, alcohol administration led to increased serum AST concentrations in female rats and reduced serum TC in male rats. Expression of the neurogenesis marker DCX decreased significantly in the hippocampi of male rats. Based on these findings, we profiled miRNAs differentially expressed in the hippocampi in response to chronic high-dose alcohol administration in male and female rats and demonstrated altered expression of 9 miRNAs including miR-125a-3p in male rats and 7 miRNAs including miR-881-3p in female rats. We identified alterations of miRNA function due to alcohol by analyzing the expression of the target genes of some miRNAs. To the best of our knowledge, our study is the first report of expression profiling of miRNAs in the hippocampi of both sexes after chronic high-dose alcohol exposure. Based on the above observations, our results suggest that chronic heavy alcohol use in humans may produce sex-dependent effects on neurogenesis and miRNA expression in the hippocampus and that sex differences should be considered when developing miRNA-based biomarkers to diagnose or treat alcoholics.

## Supplementary Information

**Additional file 1:** **Supplementary Table 1.**

**Additional file 2:** **Supplementary Table 2.** GO analysis of target genes of differentially expressed miRNAs in male rats by alcohol.

**Additional file 3:** **Supplementary Table 3.** GO analysis of target genes of differentially expressed miRNAs in female rats by alcohol.

## Data Availability

The datasets generated and analyzed in the current project are available from the corresponding author upon reasonable request.
