# Supplementary material for: Differential expression of microRNAs in the hippocampi of male and female rodents after chronic alcohol administration
Source: Biol Sex Differ. 2020 Nov 23;11:65. doi: 10.1186/s13293-020-00342-3 (PMC7684718; doi:10.1186/s13293-020-00342-3)
Supplement: Supplementary file 2 — Additional file 2: Supplementary Table 2. GO analysis of target genes of differentially expressed miRNAs in male rats by alcohol. [file 13293_2020_342_MOESM2_ESM.docx]

**Supplementary Table 2** GO analysis of target genes of differentially expressed miRNAs in male rats by alcohol

| **miRNA** | **Category** | **Term** | **Count** | ***P* value** | **Genes** |
| --- | --- | --- | --- | --- | --- |
| **Upregulated** |  |  |  |  |  |
| rno-miR-9a-5p | GOTERM_BP_DIRECT | GO:0016477~cell migration | 13 | 0.0008 | *Specc1l, Jup, Hes1, Cthrc1, Shroom2, Vav3, Cxcr4, Golph3, Pdgfrb, Enpep, Nfatc2, Lamb1, Scrib* |
|  |  | GO:0042787~protein ubiquitination involved in ubiquitin-dependent protein catabolic process | 10 | 0.0010 | *Ube2z, Maea, Rmnd5a, Nedd4, Rnf19a, Rgd1308601, Smurf2, Ube3c, Rnf146, Rnf111* |
|  |  | GO:0000122~negative regulation of transcription from RNA polymerase II promoter | 29 | 0.0016 | *Crebrf, Mef2c, Ybx3, Nfkb1, Cbx7, Cbx6, Hic1, Gata1, Myocd, Mcgf6, Otud7b, Zfp354a, Cc2d1b, Nfatc2, Klf5, Wdtc1, Asxl1, Samd11, Arid1a, Foxp1, Foxp2, Fnip2, Hes1, Nedd4, Zfp280d, Foxg1, Prdm1, Zbtb1, Tbx18* |
| rno-miR-125a-3p | GOTERM_BP_DIRECT | GO:0051567~histone H3-K9 methylation | 3 | 0.0043 | *Prdm5, Suv39h1, Baz2a* |
|  |  | GO:0048666~neuron development | 5 | 0.0058 | *Ptprz1, Map1b, Mapk9, Thoc2, Loc102551267* |
|  |  | GO:0048709~oligodendrocyte differentiation | 4 | 0.0116 | *Slc8a3, Ptprz1, Ntrk2, Nrg1* |
|  | KEGG_PATHWAY | rno04068:FoxO signaling pathway | 6 | 0.0111 | *Prkag3, Sgk1, Cdkn2b, Tgfbr1, Nlk, Mapk9* |
|  |  | rno04931:Insulin resistance | 5 | 0.0235 | *Prkag3, Rps6ka3, Mapk9, Mlxip, Ppp1cb* |
| rno-miR-98-5p | GOTERM_BP_DIRECT | GO:0000910~cytokinesis | 4 | 0.0148 | *Rab11fip4, Zfyve26, Klhl13, Ahctf1* |
|  |  | GO:0031054~pre-miRNA processing | 3 | 0.0193 | *Tarbp2, Lin28a, Lin28b* |
| rno-let-7a-5p | GOTERM_BP_DIRECT | GO:0006355~regulation of transcription, DNA-templated | 32 | 0.0016 | *E2f5, Nr6a1, Ahctf1, Hoxd1, Zfp583, Lin28a, Lin28b, Bzw1, Mycbp, Tgs1, Zfp275, Yeats4, Sox13, Tgfbr1, Arid3a, Hmga2, Hmga1, Abcg1, Loc499235, Stat2, Srebf2, Snai3, Sall4, Igsf1, Zfp282, Klhl31, Tfap2b, Pbx1, Mapk8, Apbb3, Pbx3, Rnf20* |
|  | KEGG_PATHWAY | rno04550:Signaling pathways regulating pluripotency of stem cells | 9 | 0.0028 | *Smarcad1, Nras, Pcgf3, Hand1, Onecut1, Wnt9a, Skil, Fzd4, Acvr1c* |
| rno-miR-3541 | GOTERM_BP_DIRECT | GO:0008285~negative regulation of cell proliferation | 32 | 0.0000 | *Ing4, Gabbr1, Cpeb1, Foxo4, Srf, Wt1, Fuz, Kank2, Men1, Bak1, Cdkn2a, Bcl11b, Nkx3-1, Nos3, Zfp503, Axin2, Chd5, Ptprj, Dab2ip, Nacc2, Rarg, Inppl1, Clmn, Brip1, Smad3, Sh3bp4, Cdkn1b, Jun, Bax, Nppc, Gdf11, Klf4* |
|  |  | GO:0006915~apoptotic process | 28 | 0.0002 | *Ing4, Zfp385a, Fkbp8, Hip1r, Aldoc, Eif5a, Wt1, Bak1, Pea15, Csnk2a1, Cdkn2a, Mapt, Ralb, Epo, Dab2ip, Ncf1, Hrk, Nr4a1, Pim3, Loc100125364, Ei24, P2rx1, Rnf152, Dio3, Ripk1, Bax, Six1, Nek6* |
|  | KEGG_PATHWAY | rno04310:Wnt signaling pathway | 11 | 0.0117 | *Chd8, Csnk2a1, Apc2, Jun, Vangl2, Ppp3r1, Lrp6, Mapk8, Daam1, Axin2, Tcf7l2* |
| **Downregulated** |  |  |  |  |  |
| rno-miR-324-5p | GOTERM_BP_DIRECT | GO:0048568~embryonic organ development | 3 | 0.0270 | *Pbx1, Epn2, Bmpr1a* |
|  |  | GO:0007409~axonogenesis | 4 | 0.0308 | *Klf7, Slitrk4, Lrrn2, Tnn* |
| rno-miR-181c-5p | GOTERM_BP_DIRECT | GO:0007283~spermatogenesis | 24 | 0.0003 | *Pcdha6, Pcdha7, Rad23b, Hmgb2, Rad21l1, Pcdha8, Pcdha9, Pcdha3, Hoxa11, Ube2b, Sfmbt1, Pcdhac2, Bcl2l11, Pcdhac1, Cdyl, Hmgb2l1, Pcdha10, Pcdha11, Pcdha12, Pcdha13, Usp42, Plau, Tbpl1, Il1a* |
|  |  | GO:0016567~protein ubiquitination | 22 | 0.0007 | *Anapc16, Birc6, Pcnp, Pdzrn3, Ube3c, Ube2b, Klhl2, Cul3, Pja2, Arih1, Klhl29, Trim2, Klhl5, Klhl18, Trim33, Med8, Rabgef1, Fbxo3, Rnf34, Lonrf1, Fbxl3, Tnfaip1* |
|  | KEGG_PATHWAY | rno04920:Adipocytokine signaling pathway | 6 | 0.0263 | *Irs2, Acsl1, Tnf, Prkaa1, Acsl4, Camkk1* |
