# Supplementary material for: Differential expression of microRNAs in the hippocampi of male and female rodents after chronic alcohol administration
Source: Biol Sex Differ. 2020 Nov 23;11:65. doi: 10.1186/s13293-020-00342-3 (PMC7684718; doi:10.1186/s13293-020-00342-3)
Supplement: Supplementary file 3 — Additional file 3: Supplementary Table 3. GO analysis of target genes of differentially expressed miRNAs in female rats by alcohol. [file 13293_2020_342_MOESM3_ESM.docx]

**Supplementary Table 3** GO analysis of target genes of differentially expressed miRNAs in female rats by alcohol

| **miRNA** | **Category** | **Term** | **Count** | ***P* value** | **Genes** |
| --- | --- | --- | --- | --- | --- |
| **Upregulated** |  |  |  |  |  |
| rno-miR-500-3p | GOTERM_BP_DIRECT | GO:0060174~limb bud formation | 3 | 0.0016 | *Plxna2, Sox4, Sema3c* |
|  |  | GO:0060070~canonical Wnt signaling pathway | 5 | 0.0023 | *Tbl1xr1, Loc100909913, Ndp, Sox4, Fzd4* |
|  |  | GO:0021766~hippocampus development | 5 | 0.0027 | *Slc32a1, Papd4, Pafah1b1, Nefm, Cyp19a1* |
|  | KEGG_PATHWAY | rno04360:Axon guidance | 4 | 0.0467 | *Plxna2, Sema3c, Ppp3cc, Robo2* |
| rno-miR-20b-5p | GOTERM_BP_DIRECT | GO:0016477~cell migration | 16 | 0.0013 | *Nanos1, Abi2, Rasgef1a, Arhgap35, Mmp2, Cxcl12, S1pr1, Ndel1, Tiam1, Sorbs2, Pdgfra, Jak1, Bambi, Usp24, Tnfaip1, Adam9* |
|  |  | GO:0008285~negative regulation of cell proliferation | 24 | 0.0015 | *Ptprj, Adarb1, Tsg101, Smad6, Klf11, Tgfbr2, Sox4, Rest, Rb1, Cnot7, Osm, Mfn2, Lif, Rerg, Ddr1, Tmem127, Ereg, Bcl11b, Btg3, Irf1, Rapgef2, Chd5, Tob2, Dpt* |
|  | KEGG_PATHWAY | rno04360:Axon guidance | 14 | 0.0000 | *Sema5a, Epha5, Epha4, Pak7, Limk1, Cfl2, Sema7a, Ppp3r1, Dpysl5, Sema4b, Sema3c, Dpysl2, Cxcl12, Rasa1* |
|  |  | rno04010:MAPK signaling pathway | 18 | 0.0013 | *Taok2, Tgfbr2, Ppp3r1, Mknk2, Map3k5, Dusp2, Map3k3, Elk4, Map3k2, Sos1, Map3k8, Pdgfra, Rapgef2, Map3k14, Dusp8, Map3k12, Rasa1, Map3k11* |
|  |  | rno04144:Endocytosis | 18 | 0.0023 | *Arfgap1, Rab5b, Chmp4c, Tsg101, Kif5a, Tgfbr2, Psd3, Eea1, Ldlrap1, Gbf1, Psd, Zfyve9, Rab22a, Pdgfra, Nedd4l, Rab10, Iqsec3, Rab11fip1* |
|  |  | rno04810:Regulation of actin cytoskeleton | 13 | 0.0254 | *Pak7, Fgd1, Pfn2, Tiam1, Limk1, Wasf1, Sos1, Cfl2, Ssh2, Pdgfra, Abi2, Arhgap35, Pip4k2a* |
|  |  | rno04350:TGF-beta signaling pathway | 7 | 0.0399 | *Nbl1, E2f5, Smad6, Zfyve9, Tgfbr2, Bmpr2, Bambi* |
| **Downregulated** |  |  |  |  |  |
| rno-miR-881-3p | GOTERM_BP_DIRECT | GO:0016573~histone acetylation | 4 | 0.0044 | *Supt3h, Naa50, Loc102553099, Clock* |
|  |  | GO:0000209~protein polyubiquitination | 5 | 0.0148 | *Rnf165, Rgd1308601, Ankib1, Ube2b, Cbfb* |
|  | KEGG_PATHWAY | rno04390:Hippo signaling pathway | 6 | 0.0059 | *Sox2, Prkci, Wnt6, Fgf1, Ppp1cb, Plgl2* |
| rno-miR-504 | GOTERM_BP_DIRECT | GO:0000209~protein polyubiquitination | 4 | 0.0707 | *Arih1, Rnf114, Ube2g1, Ube3c* |
|  | KEGG_PATHWAY | rno04724:Glutamatergic synapse | 4 | 0.0592 | *Grm3, Gng5, Shank3, Gng7* |
